# A Pipeline for Screening Small Molecules with Growth Inhibitory Activity against *Burkholderia cenocepacia*

**DOI:** 10.1371/journal.pone.0128587

**Published:** 2015-06-08

**Authors:** Carrie Selin, Maria S. Stietz, Jan E. Blanchard, Sebastian S. Gehrke, Sylvain Bernard, Dennis G. Hall, Eric D. Brown, Silvia T. Cardona

**Affiliations:** 1 Department of Microbiology, University of Manitoba, Winnipeg, Manitoba, Canada; 2 Department of Medical Microbiology and Infectious Disease, University of Manitoba, Winnipeg, Manitoba, Canada; 3 Department of Chemistry, University of Alberta, Edmonton, Alberta, Canada; 4 M. G. DeGroote Institute for Infectious Disease Research and Department of Biochemistry and Biomedical Sciences, McMaster University, Hamilton, Ontario, Canada; 5 McMaster High Throughput Screening Laboratory, Department of Biochemistry and Biomedical Sciences, McMaster University, Hamilton, Ontario, Canada; Ghent University, BELGIUM

## Abstract

Infections with the bacteria *Burkholderia cepacia* complex (Bcc) are very difficult to eradicate in cystic fibrosis patients due the intrinsic resistance of Bcc to most available antibiotics and the emergence of multiple antibiotic resistant strains during antibiotic treatment. In this work, we used a whole-cell based assay to screen a diverse collection of small molecules for growth inhibitors of a relevant strain of Bcc, *B*. *cenocepacia* K56-2. The primary screen used bacterial growth in 96-well plate format and identified 206 primary actives among 30,259 compounds. From 100 compounds with no previous record of antibacterial activity secondary screening and data mining selected a total of Bce bioactives that were further analyzed. An experimental pipeline, evaluating in vitro antibacterial and antibiofilm activity, toxicity and in vivo antibacterial activity using *C*. *elegans* was used for prioritizing compounds with better chances to be further investigated as potential Bcc antibacterial drugs. This high throughput screen, along with the in vitro and in vivo analysis highlights the utility of this experimental method to quickly identify bioactives as a starting point of antibacterial drug discovery.

## Introduction

*Burkholderia cepacia* complex (Bcc) is a group of Gram-negative bacteria that are widely distributed in natural and man-made environments [1]. Originally known as *Pseudomonas cepacia* [2], Bcc bacteria are characterized for their high intrinsic antibiotic resistance [3] and for their emergence as opportunistic pathogens of immunocompromised populations, including elderly people, young children, cancer patients and those with the genetic disease cystic fibrosis (CF) [4,5]. People with CF are particularly susceptible to dynamic colonization of the lung by many different microorganisms [6] but pulmonary exacerbations with Bcc are the most severe [7–9]. All species of Bcc can infect CF patients although *B*. *cenocepacia* and *B*. *multivorans* are more prevalent in North America and Europe [10]. CF patients receive aggressive antibiotic therapy early in life [11], due to the polymicrobial nature of lung infection [6]. Colonization with *Burkholderia cepacia* complex (Bcc) appears later and ranges from asymptomatic to the devastating and often fatal cepacia syndrome [12]. Besides the intrinsic antibiotic resistance of Bcc strains, antibiotic therapy options are further complicated with the emergence of multiple antibiotic resistant strains due to selection during previous antibiotic treatments [13]. Thus, developing new antibacterial molecules specifically reserved for Bcc bacteria would be desired.

Platforms widely utilized for the discovery of antibacterial drugs are high throughput screening (HTS) approaches using small molecule compound libraries to identify candidates that inhibit bacterial growth in whole cell assays or function of a key protein [14]. HTS screens for antimicrobials have also been developed using the small nematode *Caenorhabditis elegans* infected with bacteria in 96-well format. In these assays, small molecules that prolong the survival of infected *C*. *elegans* are scored automatically by automated fluorescence image analysis of worm survival, where a vital dye is taken up by dead worms [15,16]. Compounds that inhibit bacterial growth, attenuate virulence against *C*. *elegans* or enhance the worm immune system can be detected.

A large proportion of HTS studies have been executed against the major bacterial pathogens *Staphylococcus aureus* and *Mycobacterium tuberculosis* and the model organism *Escherichia coli* [17–19]. It is projected that antibiotic drug discovery in model bacteria, such as these, will produce broad-range antibiotic molecules that could potentially be used to treat a myriad of bacterial infections since antibacterial targets are typically encoded by highly conserved, bacterial essential genes [20–22]. However, many new drugs that inhibit the growth of Gram-negative *E*. *coli*, for instance, are inactive in intrinsically antibiotic resistant bacteria like Bcc [23]. Notably, there is a need to identify novel small molecules that are active in inhibiting the growth of Bcc bacteria. In this study, we describe a whole-cell based high throughput screening of a collection of approximately 30,000 small molecules [24] for growth inhibitory activity against *B*. *cenocepacia* strain K56-2. For further prioritization of the most promising hits, we illustrate a pipeline that includes antibiofilm activity and simple visual inspection of *C*. *elegans* to assess toxicity and in vivo antibacterial activity of the selected compounds.

## Material and Methods

### Bacterial strains, nematode strains, and growth conditions

*B*. *cenocepacia* K56-2, *B*. *cepacia* CEP509, *B*. *multivorans* C5393, *B*. *cenocepacia* J2315, *B*. *cepacia* C7322, *B*. *vietnamiensis* PC259, *B*. *cepacia* CEP021, *B*. *ambifaria* CEP0996, *B*. *anthina* AU1293 and *B*. *pyrrocinia* C1469 were obtained from the *B*. *cepacia* complex strain panel [25,26]. *B*. *contaminans* FFH-2055 was kindly provided by Jose Degrossi and Laura Galanternik, University of Buenos Aires and Hospital de Niños Ricardo Gutierrez. Unless indicated, Bcc strains, *E*. *coli* SY327 (Invitrogen), *Pseudomonas aeruginosa* PAO1 [27] and *Staphylococcus aureus* ATCC27700 were grown at 37°C in Luria-Bertani (LB) media. The nematode *Caenorhabditis elegans*, strain DH26, and *E*. *coli* OP50 were obtained from the Caenorhabditis Genetics Center (CGC), University of Minnesota, Minneapolis. The *C*. *elegans* strain DH26 was propagated on *E*. *coli* strain OP50 using conventional procedures [28,29].

### Drugs and small molecule libraries

The 30,259 compounds tested in the primary screening and the compounds tested during the secondary screening are from the Canadian Chemical Biology Network Compound Collection [24] (CyCC library), and their sources are described in S1 Table. The molecular weights of MAC-0151023 and MAC-0036650 were confirmed by the Manitoba Chemical Analysis Laboratory (MCAL) using electrospray ionization mass spectrometry. Briefly, a 1-μl aliquot of the resuspended compound (DMSO) was added to 1 mL of methanol and mixed thoroughly. 1 mL of a 100% methanol solution was utilized as a control, and was first injected into the electrospray ionization mass spectrometer (Varian 500-MS LC Ion Trap; Agilent Technologies) to eliminate background noise. Each of the compound samples were injected and the mass of the compounds were verified. Instant JChem 6.0.5 (http://www.chemaxon.com) was used for structure searching, chemical database access and data management.

### Primary screen

The growth inhibitory screen was performed in 96-well plate format in a final volume of 100 μL LB medium. Compounds to be tested were dissolved in DMSO and added to the plates to give a final concentration of 50 μM (5% vol/vol DMSO). Bacterial cell suspensions were prepared diluting *B*. *cenocepacia* K56-2 overnight cultures in LB to a final absorbance at 600 nm (*A*_*600*_) of 0.018. High and low growth controls consisted of 8 wells per plate containing LB with 5% DMSO with or without bacterial cell suspensions, respectively. Compounds and bacteria transfers were performed with an automated liquid handler (Biomek FX, Beckman/Coulter) and a microplate reagent dispenser (μFill, Biotek Instruments), respectively. All assay plates were incubated 5 h at 37°C in a humidified incubator, sealed with an optically pure seal, and the absorbance detector (EnVision, Perkin Elmer) was used to measure *A*_*600*_. In total, 30,259 compounds were tested in duplicate**,** with replicates on separate assay plates and data was collected in tandem.

For each assay plate, the 8 high and 8 low growth controls were used to calculate the percent residual growth (*%G*) within each well on the same assay plate as follows:

$$\%G=\left(\frac{A_{600}-\mu_{-\text{c}}}{\mu_{+\text{c}}-\mu_{-\text{c}}}\right)$$

where μ_+c_, and μ_-c_ are the *A*_*600*_ averages of the high-growth (*+c*) and low-growth (*-c*) controls.

The quality of the screen was evaluated by calculating the Z’ factor of the high- and low-growth controls according to Zhang et al. [30] using the equation

$$Z'=1-\left(\frac{3\sigma_{+\text{c}}+\text{3}\sigma_{-\text{c}}}{\mu_{+\text{c}}-\mu_{-\text{c}}}\right)$$

where σ_+c_, σ_-c,_ μ_+c_, and μ_-c_ are the standard deviations (σ) and the averages (μ) of the *% G* of the high-growth (*+c*) and low-growth (*-c*) controls. For a well-defined window, and hence a high quality screen, *Z`*should be greater than 0.5. Bioactivity was estimated by determining the B-score [31], a relative potency score calculated as the ratio of the adjusted (by median polish procedure) residual potency to the median of the absolute deviation (MAD) as follows:

$$\text{B-Score}=\left(\frac{R_{\mathrm{ijp}}}{{\mathrm{MAD}}_{p}}\right)$$

where R_ijp_ is the true measured potency without the distortion of the row (i), column (j) and plate (p) effect. MAD_p_ is the residual variability in a plate after the row and column effect are fit. The B-score normalization adjusts for any artifacts due to well location in the assay plate. B-scores with more negative values indicate compounds with higher bacterial growth inhibition activity and higher confidence. The quality control of the campaign (S2 Fig) was performed by analyzing high growth and low growth controls, which were scaled to a relative growth of 1 and 0 respectively, and had standard deviations of 0.043 and 0.014 respectively (S2A Fig). Overall, the screening window was sufficient with a Z’ value of 0.83 and the replicates of residual growth R1 and R2 correlated well with each other (S2B Fig).

### Secondary screen

One hundred compounds selected from the primary screen were reassayed at a final concentration of 50 μM and 5% vol/vol DMSO in 96-well format. *B*. *cenocepacia* K56-2 was cultured overnight in LB broth, diluted to an A_600_ of 0.018 in LB and 95μl of the cell suspensions were added into 96 well plates containing the compounds to be tested in four replicates. Positive and negative control wells consisted of LB with DMSO (5% v/v) with or without bacterial cell suspensions, respectively. All plates were sealed, incubated at 37°C for 5 h, and after incubation, A_600_ was recorded after shaking for 15 seconds in a BioTek Synergy 2 plate reader. For each assay plate the residual growth was determined as the A_600_ in the presence of the tested compound/A_600_ in the absence of the compound.

### Determination of minimal inhibitory concentration (MIC) and minimal bactericidal concentration (MBC)

The standard microtitre broth dilution method as specified by CLSI guidelines [32] in Mueller Hinton Broth (MHB) with cation supplementation (CAMHB) was used for MIC determination with a final inoculum of 10^5^ cfu/mL. Microtitre plates were read after 22 h incubation at 37°C. As shown in S5 Table, the highest soluble test concentration for each compound ranged from 512 μg/mL, 128 μg/mL or 64 μg/mL. Of the 49 Bce bioactives tested 11 were unable to solubilize at any concentration and were therefore not tested. To determine the MBC, 100 μL aliquot from wells for which no significant difference in optical density was observed between the inoculated and blank wells, was plated onto LB agar and visually inspected after 48 h of incubation. The MBC was defined as the lowest concentration for which no colony forming units (CFU) were observed after incubation. For all MICs and MBCs two independent experiments were performed. Results between experiments never differed by more than two-fold. In case a two-fold difference was observed, the highest value was considered.

### Minimal biofilm inhibitry concentration (MBIC) determination

The MBIC was determined by resazurin-based viability staining as previously described [33]. Overnight cultures were diluted in CAMHB at a final inoculum of 10^5^ cfu/mL. A hundred microliters of the diluted cell suspensions were transferred to the wells of a polystyrene round-bottomed 96-well microtiter plate (SARSTEDT AG & Co, Germany) and incubated at 37°C. After 4 h of adhesion, the supernatant was removed and the plates were rinsed twice with physiological saline solution (PS, 0.85% NaCl). Subsequently, 100 μL of antibiotic-containing CAMHB (using antibiotic concentrations identical to those in the MIC experiments) or 100 μL PS (control) was added at each well and plates were further incubated at 37°C. After 20 h of treatment, plates were again rinsed twice with PS and the presence of metabolically active sessile cells was detected with a commercially available resazurin solution (CellTiter-Blue, CTB, Promega, Madison, WI, USA). A hundred microliters of PS and 20 μL of resazurin were added to each well, plates were incubated for 1 hour at 37°C and finally fluorescence was measured using a BioTek Synergy 2 plate reader (λ_ex_, 530 nm; λ_em_, 590 nm) [33]. All MBIC experiments were performed in duplicate.

### Hemolytic assay

The hemolytic activity of the compounds displaying an MIC against *B*. *cenocepacia* K56-2 was determined as previously described [34] with some amendments. Briefly, 500 μL of erythrocytes (sheep red blood cells (RBC) Alere, Canada) were washed three times in phosphate buffered saline (PBS) (per 100 mL: 0.8 g NaCl, 0.02 g KCl, 0.144 g Na_2_HPO_4_, 0.024g KHPO_4_, pH 7.4) and resuspended in a final volume of 5 mL with PBS to give a working suspension of 10% RBC. 200 μL of the 10% RBC suspension was incubated for 1 hour with 50, 100, 500 and 1000 μg/mL of test compound dissolved in PBS and DMSO. MAC-0000212, MAC-0164811, MAC-0170906, MAC-0040413, MAC-0040599, MAC-0044103 and MAC-0046591 were assayed at concentrations of 25–250 μg/mL and MAC-0036650 was assayed at concentration of 25–50 μg/mL due to solubility issues in PBS. The samples were centrifuged (4000 rpm, 5 min) and placed on ice prior to measuring the absorbance of the supernatant at 540 nm. PBS and 0.1% Triton X-100 were used as negative and positive controls respectively. A range of 10–0.5% DMSO in PBS was also used as a control. Percent hemolysis was calculated using the following formula:

$$\%\;\mathrm{hemolysis}=\frac{{\mathrm{OD}}_{540}-{\mathrm{OD}}_{540}\text{DMSO control}}{{\mathrm{OD}}_{540}\;\text{TritonX}-100}\times 100$$

Two independent experiments were carried out in triplicate. A high and low percent hemolysis for a given compound was categorized within the range of > 40% and 5–10%, respectively [35].

### Liquid killing assays

In vivo antibiotic activity of compounds that illustrated an MIC against *B*. *cenocepacia* K56-2 were tested on *C*. *elegans* using the liquid killing assay (LKA) described by Kaplan et al [36]. Briefly, eggs of *C*. *elegans* DH26 (obtained from the *Caenorhabditis* Genetics Center) were hatched on lawns of *E*. *coli* OP50 on nematode growth medium agar. Worms were grown to L4 stage by incubation at 25°C for 48 h. Worms were washed off the plate with M9 buffer, deposited onto lawns of *E*. *coli* OP50 or *B*. *cenocepacia* K56-2 and the plates were incubated at 25°C for 16 h. OP50-fed or *B*. *cenocepacia*-infected worms were washed from the plate with M9 buffer, allowed to settle to bottom of Eppendorf tubes, and rinsed with M9 buffer. The M9 was then removed and worms were resuspended in 5 mL liquid killing medium (80% M9 and 20% NGMII) and 95 μL aliquots with approximately 20–30 worms were added to the wells of 96-microtiter plates with or without test compounds at the indicated concentrations calculated as fold-MICs. As a control, worms treated without test compound contained an equivalent amount of DMSO in the assay medium. We also tested four antibiotics known to inhibit the growth of *B*. *cenocepacia* K56-2; trimethoprim (TP; 10 μg/mL), meropenem (Mero; 32 μg/mL), chloramphenicol (Chl; 10 μg/mL) and tetracycline (Tet; 4 μg/mL). Plates were scored for live worms at the time of inoculation and every 24 h for 6 days using a dissecting microscope. Worms were considered dead if they appeared straight, and alive if they appeared S-shaped and were moving. The percentage of nematode survival was calculated via the Kaplan-Meier method and plotted as the percent survival as a function of time. The survival kinetics were compared by using nonparametric log-rank test and were considered statistically different when P was <0.05.

### Survival 100 (Surv_100_) assay, and Surv_100_/MIC ratio determination

An initial LKA was carried out as described above, with some modifications. Five to 10 OP50-fed worms per well were exposed to each compound, and serially diluted from its highest soluble concentration. The following concentration range was tested for each compound: MAC-0041192: 32–0.03 μg/mL; MAC-0036650, MAC-0040158, MAC-0040413 and MAC-0040599: 64–0.06 μg/mL; MAC-0000212 MAC-0044103 MAC-0046591, and MAC-0164811: 128–0.125 μg/mL; MAC-0013209, MAC-0151023, MAC-0164385 and MAC-0168816: 512–0.25 μg/mL. Worms were counted at 0 hours of exposure and once again at 24 hours. The highest concentration where 100% nematode survival was observed at 24 hours was designated Surv_100._ Percent survival of the nematodes was determined for each of the test compounds at each concentration and the ratio of Surv_100_/MIC was calculated. The Bce bioactives that demonstrated a ratio of 1 and greater was used in the in vivo antibiotic activity assay. The toxicity and the Surv_100_/MIC’s of known antibiotics Chloramphenicol (Chl), Tetracycline (Tet), Trimethoprim (Tp) and Meropenem (Mero) were also evaluated as described. The concentration range for Chl, Tet, and Tp was 1 to 1000 μg/mL, while the concentration range used for Mero was 0.5 to 500 μg/mL.

## Results

### Screening for growth inhibitors of *B*. *cenocepacia* K56-2

As there are no previous records of high throughput screens using Bcc bacteria, we ran and analyzed a pilot screening campaign of 480 compounds in duplicate using *B*. *cenocepacia* K56-2, as previously described for the *E*. *coli* strain MC1061 [37]. To increase the sensitivity of the screen we measured growth inhibition at 5 hours, which corresponds to actively replicating *B*. *cenocepacia* cells. Z’ values of 0.82 and 0.81 (S2 Table) for first and second replicates, respectively indicated a high quality of the screen for high-throughput purposes. Setting the threshold for the hit cut off at 3 standard deviations of the high growth controls yielded 2 active compounds, which represents a hit rate of 0.42% (S1 Fig); thus we anticipated that approximately 125 active compounds should be found with a screen of the CyCC library.

**Fig 1:**
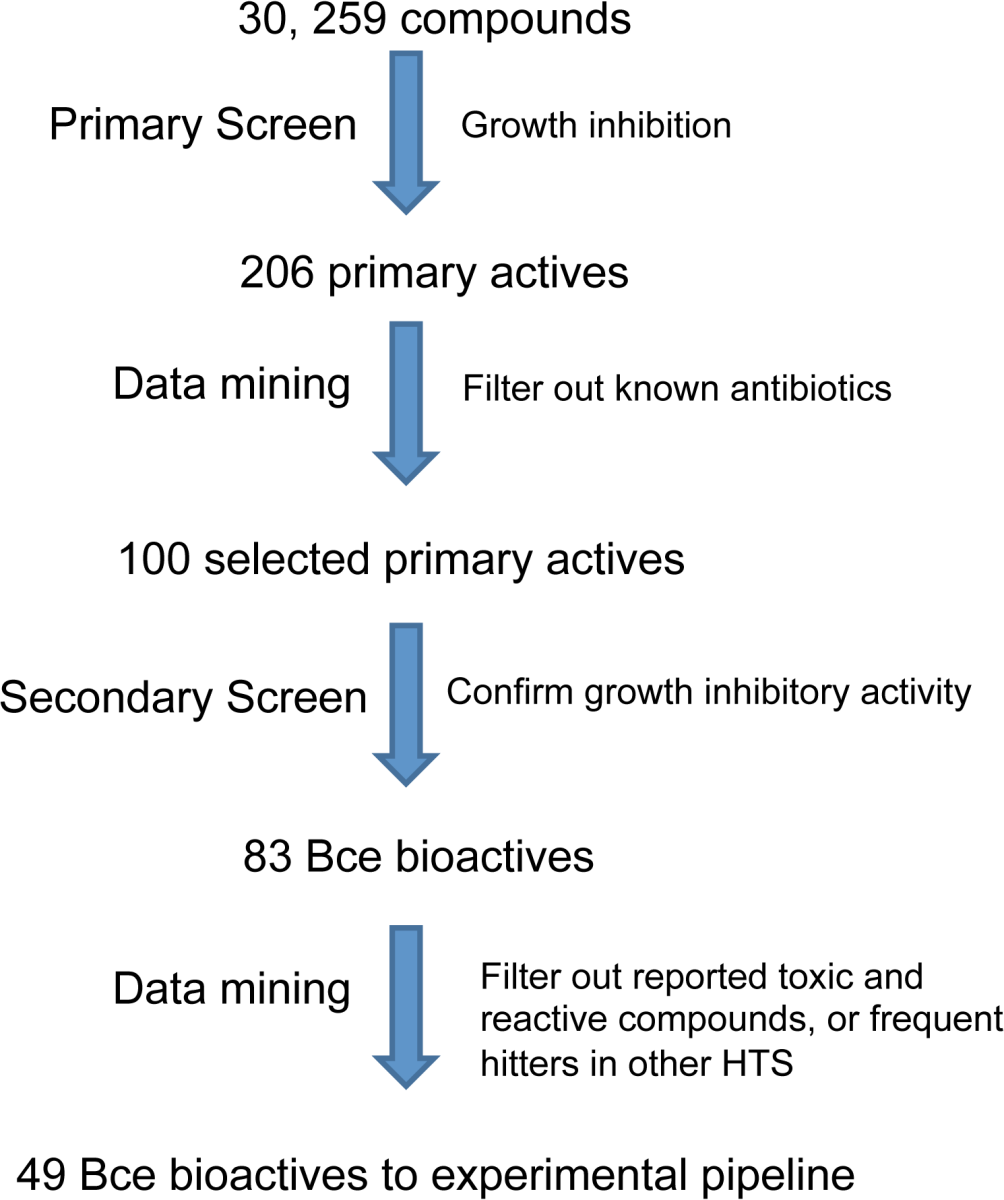
High throughput screening and compound prioritization process. Steps are shown as arrows with the name of the step to the left and the selection method to the right.

**Fig 2:**
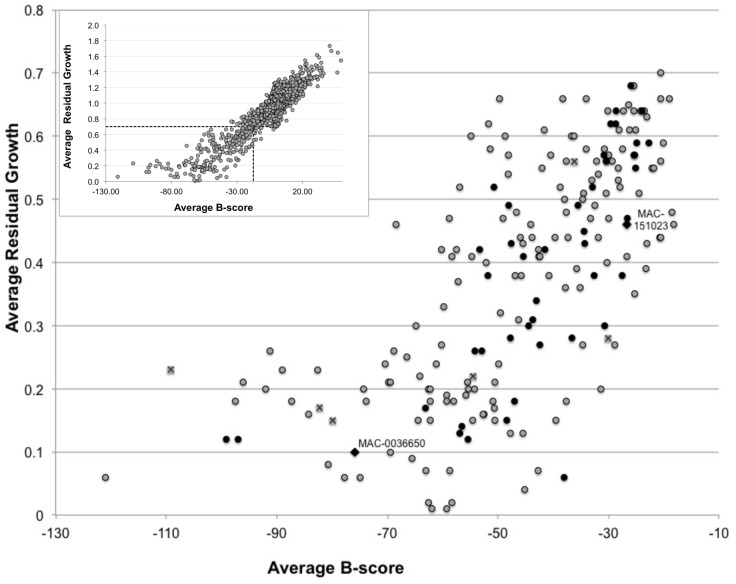
Overview of the primary screen results. The average residual growth of each compound is plotted against the Average B-score. The inset shows the application of an average residual growth cutoff of 0.70 and a B-Score cutoff of –17.5, (dashed line) to the whole screening campaign, which identified 206 Bce bioactives. The large scatter plot shows the 206 primary actives. For comparison reasons, 6 known antibiotics, are denoted with crossed rectangles, ceftriaxone sodium, (-121, 0.06); minocycline, (-109, 0.23); metampicilin, (-80.7, 0.08); chloramphenicol, (-68.8, 0.26); ciprofloxacin, (-35, 0.36); trimethoprim, (-29.6, 0.62). The two Bce bioactives with the most promising properties are indicated with diamonds and labeled.

The screening and selection process of compounds is outlined in Fig 1. The first step or primary screen started with 30,259 compounds (S1 Table) analyzed in a single dose of 50 μM against *B*. *cenocepacia* K56-2 growth. To identify active compounds, we calculated the statistical cut off rate as 3 standard deviations from the mean (i.e., 1-3sigma, where sigma was 0.98) for the full set of tested compounds. This cut off rate of 0.70 identified 222 primary actives. The average B-score was scaled to zero with a threshold to identify active compounds defined as 1-3X the standard deviation of the data or –17.5. Application of both thresholds to identify active compounds (<0.70 residual growth and <–17.5 B-Score,) selected 206 primary actives (Fig 2).

We first focused on the 20 compounds with the highest B-Score (S3 Table). Included were 3 imido-piperidines previously synthesized by Hall et al [38] and MAC-0036650, a synthetic phenyl-thiazol-butenamide from the Maybridge collection. The rest of the compounds corresponded to known antibiotics. Overall, the 206 primary actives included 97 hits previously described as antibiotics or disinfectants, indicating that the screening for growth inhibitors selects for small molecules of antibacterial action. These known antibiotics with the exception of 4 that were used as controls were excluded from further analysis. Thus, 113 primary actives were initially selected for secondary screening; due to lack of availability of 13 compounds at the time of the screening, 100 compounds were finally tested for growth inhibition. The secondary screening measured residual growth in the presence of 50 μM drug concentrations. Compounds that caused residual growth equal or less than 0.85 were selected and termed Bce bioactives. We searched the PubMed Compound [39] and PubMed BioAssay [40] databases to filter out compounds with reported toxicity, reactivity and frequent hitters found in other HTSs. This allowed for a total of 49 Bce bioactives to be selected for further analysis (S4 Table).

### Analysis of the minimum inhibitory concentration (MICs) of the novel compounds

Due to solubility issues, 39 of the 49 compounds were subjected to standard MIC determination, according to CLSI guidelines using *B*. *cenocepacia* K56-2 (S6 Table). A total of 15 compounds exhibited detectable MICs against *B*. *cenocepacia* K56-2 (Table 1). Six compounds, MAC-0036650, MAC-0040599, MAC-0041192, MAC-0044103, MAC-0151023, and MAC-0164811 showed MIC values equal to or lower than 32 μg/mL, which was similar to the MIC value for meropenem. When the 15 compounds were tested against ten of the Bcc panel strains, MAC-0164811 and MAC-0044103 also showed MIC values ≤ 32 μg/mL in seven and six of the tested strains, respectively (Table 2). MAC-0040599, MAC-0041192 and MAC-0151023 illustrated activity (MIC values ≤ 32 μg/mL) against four of the Bcc strains assayed. In addition, *E*. *coli*, and *P*. *aeruginosa* were used in MICs determinations (S6 Table) as we reasoned that compounds that were active against *B*. *cenocepacia* might be also active against *E*. *coli* and *P*. *aeruginosa*. With the exception of MAC-0000212, the compounds were indeed active against *E*. *coli*. In general, *E*. *coli* seemed to be more susceptible to the majority of the compounds tested. On the contrary, the only compounds that were moderately active against *P*. *aeruginosa* were MAC-0040599, MAC-0041192 and MAC-0183697, with MICs values of 64 μg/mL. With the exception of MAC-0000212, MAC-0036650, MAC-0040413 and MAC-0040599 the compounds that were active against *B*. *cenocepacia* K56-2 also inhibited *S*. *aureus* (S6 Table). When the antibiofilm effect of the compounds was assayed against 4 h old biofilms, MAC-0036650 and MAC-0040599 showed MBIC values equal to or lower than 64 μg/mL against *B*. *cenocepacia* K56-2 and five strains from the Bcc strain panel. MAC-0044103 and MAC-0164811 were the most bactericidal compounds with MBC values ≤ 64 μg/mL in nine and six of the Bcc strains, respectively (Table 2).

**Table 1:** Minimum inhibitory concentration (MIC) of the top 15 synthetic compounds against *B*. *cenocepacia* K56-2, the Survival_100_ (Surv_100_) concentration in *C*. *elegans* and the Surv_100_/MIC ratio.

Cnd_ID	MIC (μg/mL)	SURV_100_ (μg/mL)	SURV_100_/MIC
MAC-0000212	64	16	0.25
MAC-0004910	256	64	0.25
MAC-0013209	128	256	2
MAC-0031247	256	128	0.5
MAC-0036650	16	32	2
MAC-0040158	64	4	0.06
MAC-0040413	64	2	0.03125
MAC-0040599	32	8	0.25
MAC-0041192	32	4	0.125
MAC-0044103	32	8	0.25
MAC-0046591	128	64	0.5
MAC-0151023	32	128	4
MAC-0164385	512	16	0.03
MAC-0164811	16	8	0.5
MAC-0168816	256	4	0.01
Trimethoprim	10	1000	100
Chloramphenicol	10	1000	100
Tetracycline	4	1000	250
Meropenem	32	500	15.6

^a^MIC was determined using the standard microtitre broth dilution method as specified by CLSI guidelines [32] in MHB with cation supplementation and a final inoculum of 10^5^ CFU/mL. The concentration range of the test compounds for the MIC is listed in S5 Table.

^b^ The Surv_100_ was defined as the highest soluble compound concentration that results in 100% survival (SURV_100_) of *C*. *elegans* within a 24 hour time frame_._ The Surv_100_ assay was conducted as an LKA where 5 to 10 OP50-fed worms per well were exposed to each compound, serial diluted from its highest soluble concentration, for 24 hours at 25°C. The % survival was determined and the ratio of Surv_100_/MIC was calculated. The compounds with a ratio of 1 and greater were used in the in vivo antibiotic activity assay.

**Table 2:** MICs, MBCs and MBICs for the top 15 compounds tested against ten Bcc strains^a^.

Compound	MIC range (μg/mL)	N° of strains with MIC ≤ 32 μg/mL	MBC range (μg/mL)	N° of strains with MBC ≤ 64 μg/mL	MBIC range (μg/mL)	N° of strains with MBIC ≤ 64 μg/mL
MAC-0000212	16 - >128	3	64 - >128	2	64 - >128	2
MAC-0004910	512 - >512	0	512 - >512	0	256–512	0
MAC-0013209	64–256	1	128–256	0	128–512	0
MAC-0031247	128–512	0	512 - >512	0	256 - >512	0
MAC-0036650	8 - >64	3	32 - >64	4	64 - >64	5
MAC-0040158	32–512	1	64–512	2	64–512	1
MAC-0040413	32 - >64	2	64 - >64	2	32 - >64	5
MAC-0040599	16–64	4	64 - >64	5	32 - >64	5
MAC-0041192	32–128	4	32–512	3	64–256	2
MAC-0044103	16–64	6	8–128	9	64–128	4
MAC-0046591	128 - >128	0	>128	0	>128	0
MAC-0151023	16–128	4	512 - >512	0	64–256	1
MAC-0164385	32–256	2	128–512	0	64–256	1
MAC-0164811	4–64	7	32–128	6	32–128	7
MAC-0168816	128–512	0	256 - >512	0	256 - >512	0

^a^ The strains tested were *B*. *cepacia* CEP509, *B*. *multivorans* C5393, *B*. *cenocepacia* J2315, *B*. *cepacia* C7322, *B*. *vietnamiensis* PC259, *B*. *cepacia* CEP021, *B*. *ambifaria* CEP0996, *B*. *anthina* AU1293, *B*. *pyrrocinia* C1469 and *B*. *contaminans* FFH-2055.

### Toxicity analysis

**Fig 3:**
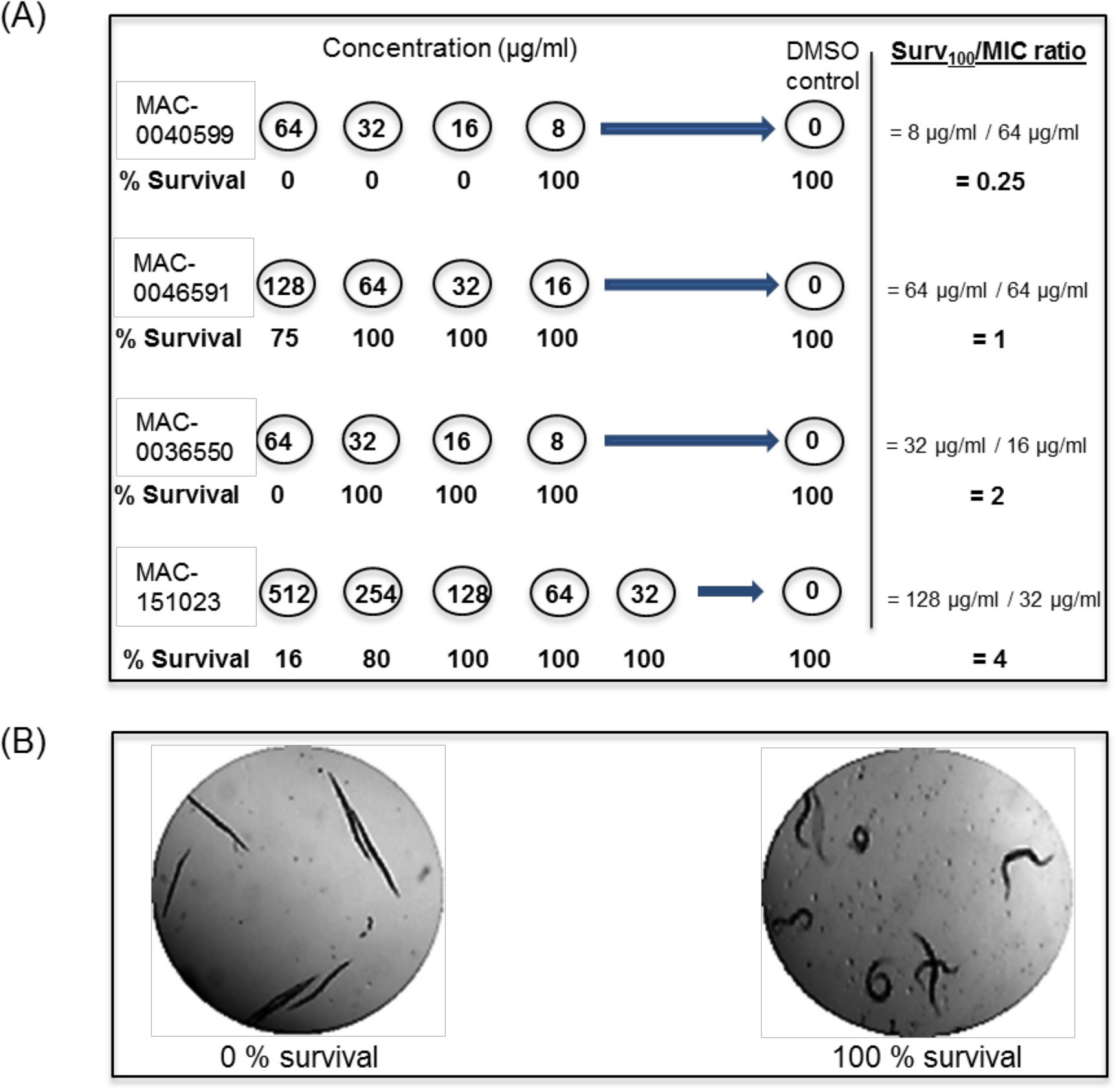
Survival _100_ (Surv_100_) assay, and the Surv_100_/MIC ratio determination. (A) The Surv_100_ assay was conducted in a 96-well format, where each compound was serially diluted along the rows from its highest soluble concentration or the MIC. The last well for each compound was used as a DMSO control. Approximately, 5 to 10 OP50-fed worms were added to the wells containing LKM for a total assay volume of 100 μl. The number of worms was counted and recorded for each concentration on day 0 and again 24 hours later. Percent survival was determined for each concentration. The Surv100/MIC ratio was calculated. The compounds, which demonstrated a ratio of 1 and greater, were used in the in vivo antibiotic activity. (B) Photograph of the worms from the assay illustrating the difference between 0% survival and 100% survival.

Hemolysis experiments with sheep red blood cells (RBC) were performed to investigate possible membrane-disturbing activity of the compounds against eukaryotic cells. At the concentration tested (50 to 1000 μg/mL; 25–250 μg/mL; 25–50 μg/mL) the compounds did not show induced hemolysis of RBC (Table 3); however, at 1000 μg/mL MAC-0013209 illustrated higher hemolysis than most compounds at 11.5%, which is moderately hemolytic. Even though the compounds did not exhibit hemolytic activity, it was observed that some of the compounds caused 100% death in non-infected *C*. *elegans* within 24 hours. For this reason, we utilized an initial toxicity screen in the host model *C*. *elegans*, designated as the Surv_100_ assay, to assess the toxicity of a given compound towards non-infected nematodes after 24 hours of exposure. As exemplified in Fig 3A, a range of 11 concentrations were tested for the ability to cause death in the worms. Exposure to the highest tested concentration for each of the compounds, with the exception of MAC-0151023, resulted in 100% death in *C*. *elegans*. When we examined the compound concentrations that permitted 100% nematode survival (Surv_100_), the bioactive concentration range was from 2–256 μg/mL (Table 1). Many compounds illustrated Surv_100_ concentrations that were lower than the concentrations needed to inhibit *B*. *cenocepacia* K56-2 (Table 1). As a result, when we calculated the Surv_100_/MIC ratio for each these compounds, the value was less than 1 (Table 1). In contrast, MAC-0013209, MAC-0036650, and MAC-0151023 displayed Surv_100_ concentrations that were higher than their respective MICs, and as such, exhibited a Surv_100_/MIC ratio greater than 1 (Table 1). Instead the antibiotics Tp, Tet, Chl, and Mero, used for comparison, illustrated a Surv_100_ concentration in the range of 500–1000 μg/mL (Table 1). Since this is substantially larger than the respective MICs, the calculated Surv_100_/MIC ratio ranged from 15.6–250 (Table 1).

**Table 3:** Hemolytic activity (% hemolysis^*^) of the bioactive compounds that inhibit the growth of *B*. *cenocepacia* K56-2.

**μg/mL**	**MAC-0013209**	**MAC-0037247**	**MAC-0040158**	**MAC-0041192**	**MAC-0151023**	**MAC-0164385**	**MAC-0168816**	**MAC-0171207**
1000	11.50 (1.85)	5.32 (0.87)	6.23 (1.29)	7.30 (1.50)	7.32 (0.91)	8.08 (0.78)	1.70 (0.62)	2.64 (0.53)
500	7.45 (1.15)	3.21 (1.20)	1.30 (0.52)	0.90 (0.20)	2.06 (0.07)	2.46 (0.14)	1.07 (0.14)	1.32 (0.70)
100	2.72 (0.89)	1.53 (0.32)	0.69 (0.30)	0.50 (0.60)	1.00 (0.08)	1.03 (0.18)	0.90 (0.05)	0.53 (0.26)
50	1.22 (0.95)	0.41 (0.95)	0.35 (0.15)	0.24 (0.13)	0.27 (0.03)	0.11 (0.10)	0.09 (0.08)	0.35 (0.15)
**μg/mL**	**MAC-0000212**	**MAC-0036650**	**MAC-0040413**	**MAC-0040599**	**MAC-0044103**	**MAC-0046591**	**MAC-0164811**	
250	2.34 (0.45)		2.12 (0.34)	3.21 (0.23)	1.85 (0.44)	9.5 (2.2)	5.53 (0.02)	
100	1.33 (0.85)		1.13 (0.32)	2.02 (0.56)	0.75 (0.04)	7.95 (1.64)	2.33 (0.01)	
50	0.99 (0.12)	3.25 (1.02)	0.51 (0.05)	1.01 (0.13)	0.63 (0.21)	1.75 (0.59)	2.18 (0.03)	
25	0.24 (0.04)	0.98 (0.29)	0.15 (0.10)	0.45 (0.33)	0.13 (0.04)	0.32 (0.11)	0.36 (0.02)	

Hemolytic activity of each compound was determined by incubating 50, 100, 500 and 1000 μg/mL of test compound with 200 μl of a 10% RBC suspension for 1 hour at 37°C. PBS and 0.1% Triton X-100 were used as negative and positive controls respectively. A range of 10–0.5% DMSO in PBS was also used as a control. Note that MAC-0000212, MAC-0040413, MAC-0040599 and MAC-0044103, MAC-0046591, and MAC-0164811, were assayed using a range of 25–250 μg/mL, while MAC-0036650 was assayed at a range of 25–50 μg/mL.

^*^% Hemolysis values are the average (Std Dev) of two independent experiments performed in triplicate. A high and low percent hemolysis for a given compound was categorized within the range of > 40% and 5–10%, respectively [35]

### *In vivo* antibiotic activity in *C*. *elegans* infected with *B*. *cenocepacia* K56-2

**Fig 4:**
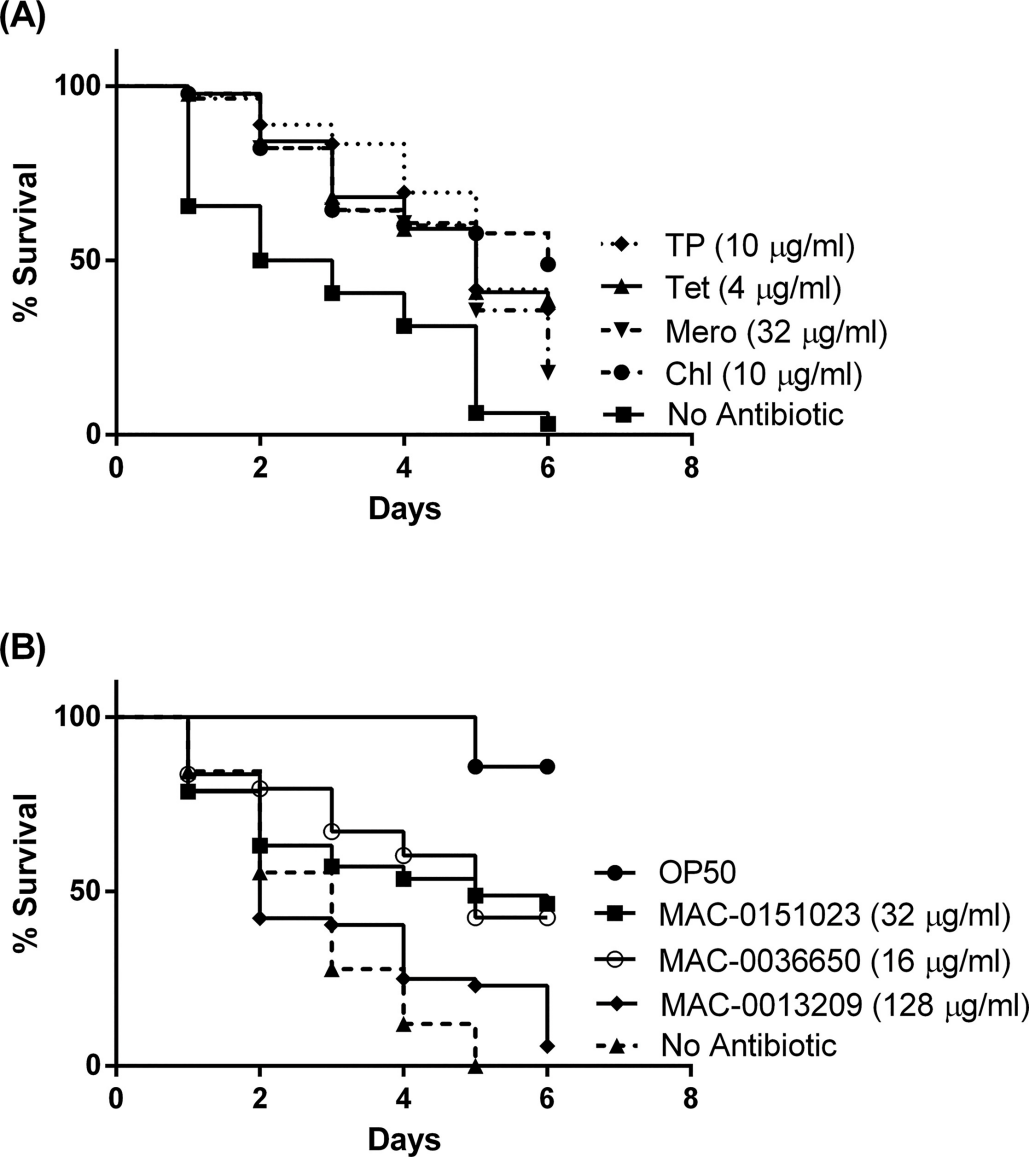
*C*. *elegans* rescue assays. *C*. *elegans* was allowed to feed on *B*. *cenocepacia* K56-2 and OP50 for 16 hours. The *B*. *cenocepacia* infected and OP50-fed worms were subsequently treated with (A) Trimethoprim (TP), Tetracycline (Tet), Meropenem (Mero), Chloramphenicol (Chl) and observed for 6 days every 24 hours and were compared to the non-treated (No Antibiotic) worms for survival. p < 0.0001 for all compounds. (B) The worms were treated with MAC-0013209, (p = 0.2503) with MAC-0151023 and MAC-0036650 at their respective MIC (128 μg/mL, 32 μg/mL and 16 μg/mL); p< 0.0001 for both MAC-0151023 and MAC-0036650 compounds. Trimethoprim was used as a control. p < 0.0001 for all concentrations.

We chose MAC-0013209, MAC-0036650 and MAC-0151023 for in *vivo* assessment of antibacterial activity in *C*. *elegans* as these compounds observed the highest Surv_100_/MIC ratios among the investigated bioactives. L4 nematodes were infected with *B*. *cenocepacia* and moved to 96-well plates in the presence and absence of the compounds at a concentration of the MIC. Our initial *in vivo* antibiotic activity assay carried out with Tet, Mero, Chl, and TP, illustrated the ability of these compounds to rescue *B*. *cenocepacia* infected worms (Fig 4A). Similarly, we detected that nematodes died much more quickly in the absence of the tested Bce bioactives than in their presence (Fig 4B). The treatment with the compounds MAC-0151023, and MAC-0036650 at their respective MICs, prolonged the survival of the infected worms by approximately 40–50% compared to the non-treated control (p <0.0001 for both; Fig 4B). MAC-0013209, however had no effect in prolonging survival of the worms (Fig 4B). At higher concentrations, the positive effect exhibited by MAC-0151023 and MAC-0036650 was reduced (data not shown). This was not the case when worms were treated with higher concentrations of Tp, where the positive effect exhibited by the antibiotic was not reduced in any way (data not shown).

## Discussion

The aim of this study was to identify *B*. *cenocepacia* K56-2 inhibitory compounds via a whole cell based high throughput screening and to develop a simple pipeline for prioritizing compounds with a better chance to be further investigated as antimicrobial leads. This process utilized visual inspection of *C*. *elegans* survival for evaluation of toxicity and in vivo antibacterial activity. The experimental screening and selection pipeline of our HTS identified a total of 49 *B*. *cenocepacia* inhibitory compounds (S5 Table). Only 39 of the compounds were soluble in CAMHB medium (S5 Table; Table 1), and as a result, the in vivo antibacterial activity could not be evaluated for all of the compounds. Out of the 39 compounds that were tested, 15 of the Bce bioactives displayed detectable MICs against *B*. *cenocepacia* K56-2 (Table 1); 6 of which exhibited an MIC ≤ 32 μg/mL. Once again, MICs could not be obtained for many of the compounds due to insolubility at higher concentrations in CAMHB media (data not shown). Evidently, compound solubility plays a large role in the assessment of antibacterial activity. It has been shown that nearly 20–30% of compounds within chemical collections are not soluble at low DMSO concentrations of 10–30 mM [41–43], which explains why when conducting the MIC assay, at a DMSO concentration of 10 mM, approximately 70% of the Bce bioactives that were under investigation precipitated from solution and could not be efficiently measured. A simple solution would be to increase the DMSO concentration to enhance compound solubility; however, DMSO concentrations that exceed 10 mM significantly affect the growth of *B*. *cenocepacia* K56-2 and would reduce the efficacy of the MIC assay.

With a total of 15 Bce bioactives displaying an MIC against *B*. *cenocepacia* K56-2, we then wanted to determine if any of these compounds displayed toxicity, as it is a commonly known feature among chemical compounds within collection libraries [14]. None of the compounds exhibited hemolytic activity when we assessed their ability to lyse sheep red blood cells. However, it remained a possibility that these compounds could display toxicity towards other organisms. Previous studies have shown that the fecundity of *C*. *elegans* can be used for assessing toxicity of many compounds, including heavy metals, environmental pollutants, organic solvents and neurotoxins [44,45]. These assays involve incubating worms for many days in the presence of the compounds and observing progeny number. Instead, we measured toxicity by visual scoring of the typical shape of dead worms after 24 h. With this method, trimethoprim, chloramphenicol, tetracycline and meropenem were scored as non-toxic while the Bce bioactives displayed various levels of toxicity. This illustrate that the assay can discriminate between non-toxic and toxic compounds. By monitoring worm survival upon compound exposure and calculating a Surv_100_/MIC ratio for each of the compounds, we were able to estimate a toxic/effective ratio for each of the Bce bioactives. With this strategy, 12 out of the 15 compounds illustrated Surv_100_/MIC ratios substantially lower than 1, were lethal towards the nematodes at their respective MICs (Table 1) and were eliminated from additional examination. Three of the compounds, MAC-0013209, MAC-0036650, and MAC-0151023, exhibited a ratio ≥1 (Table 1), were not lethal to the worms at their relevant MIC, and were selected for further analysis. Evidently, by using *C*. *elegans* and numerically evaluating compound toxicity via the Surv_100_/MIC ratio, the Bce bioactives that displayed low levels of toxicity were easily determined. When we calculated the Surv_100_/MIC ratios for Tp, Mero, Chl, and Tet, the ratios ranged between 15 and 250 (Table 1), which is approximately 4 to 60 times greater than the highest ratio of 4 obtained for MAC-0151023 (Table 1). The elevated ratio values obtained for these antibiotics was not surprising, as Tp, Mero, Chl, and Tet were not lethal towards *C*. *elegans* at concentrations ranging from 1 μg/mL to 1000 μg/mL. Moreover, they displayed strong growth inhibition towards *B*. *cenocepacia* K56-2 with MIC values ranging from 4 μg/mL to 32 μg/mL (Table 1). A different trend was observed for MAC-0013209, MAC-0036650, and MAC-0151023, where compound toxicity was only seen at the highest soluble concentration and they exhibited a broader MIC range of 16 μg/mL to 128 μg/mL (Table 1). Structurally, the bioactive compounds are as diverse as Tp, Mero, Chl, and Tet, in terms of their R-groups, carbon chains, and aromatic ring structures; however, it has been shown that small differences play a large role in the compounds physiochemical properties. These properties determine how active a drug is in terms of being able to reach its target and bind to the adequate sites [46].

From our Surv_100_/MIC ratios, we identified three compounds, MAC-0013209, MAC-0036650, and MAC-0151023, which displayed low toxicity at their respective MICs. In order to determine if these compounds displayed in vivo antibacterial activity, we tested each of the compounds for their ability to prolong survival in *B*. *cenocepacia* K56-2 infected-*C*. *elegans*. Only 2 of the compounds, MAC-0151023 and MAC-0036650, displayed the ability to extend worm survival at their respective MICs (Fig 4B). Interestingly, at higher concentrations, the positive effect exhibited by MAC-0151023 and MAC-0036650 was abolished (data not shown). The observation that compounds are less active in rescuing *C*. *elegans* from infection at higher concentrations has been reported previously for fluconazole [47], where the authors suggested that the diminished capacity of fluconazole to rescue infected worms at a higher concentration is likely due to toxicity. We previously determined in our toxicity assay that the higher concentration of MAC-0151023 (128 μg/mL) and MAC-0036650 (32 μg/mL) were not toxic to non-infected OP50-fed nematodes as 100% of the worms survived exposure at these concentrations (SURV_100_; Table 1). Furthermore, the *in vivo* antibacterial assays also indicated that prolonged exposure to the compound at these concentrations were not lethal to OP50-fed worms (Fig 4B). Because the study conducted by Breger et al (2007) did not determine the effect of fluconazole on non-infected worms, we can only conclude that it appears as though the reduction in anti-infective activity of the bioactive compound may be due to the lethal combination of infected *C*. *elegans* in conjunction with exposure to higher levels of the test compound.

In summary, after a HTS of more than 30,000 small molecules, and selection of compounds with in vitro antibacterial and antibiofilm activity, we were quickly able to prioritize compounds of lower toxicity and better antibacterial activity using *C*. *elegans* as a host model. We then propose that our experimental pipeline can be effectively applied to antibiotic drug discovery efforts against any microorganism capable of infecting *C*. *elegans*.

## Supporting Information

S1 Fig A pilot screening campaign of 480 small molecules for growth inhibitors of *B*. *cenocepacia* K56-2. Compounds were tested at 50 μM and 5% DMSO in 96-well format in a final volume of 100 μL. Bacteria was added as a cell suspension obtained from an overnight culture diluted to *A*_*600*_ of 0.018 in LB. High and low growth controls (8 wells per plate) contained bacteria in LB with 5% DMSO or LB with 5% DMSO, respectively. All assay plates were incubated at 37^°^C in a humidified incubator. After 5 hr, plates were removed from incubation, sealed with an optically pure seal, and *A*_*600*_ was measured for 15 s within the absorbance detector (EnVision, Perkin Elmer). In total, 480 compounds were tested in duplicate, with replicates on separate assay plates prepared, and data collected, in tandem.
(PDF)

S2 Fig Primary screen quality control. A. *B*. *cenocepacia* K56-2 growth in 5% DMSO (high growth controls, circles) were scaled to a residual growth value of 1. Low growth controls (squares) represent media with no bacteria. Controls were run in replicates and replicate 1 (clear symbols) and replicate 2 (Grey symbols) are shown. B. Scatter Plot of the CYCC Library primary screen against *B*. *cenocepacia* K56-2 growth Correlation between replicate (R1) and replicate 2 (R2) is shown. Grey circles represent compounds. The statistical cut off rate to identify active compounds was 0.87, as calculated as 1- 3X standard deviation of the high controls, shown as a solid line. This identified 774 actives, a large number of compounds to initially follow up. One method by which to initially focus on the most potent actives is to set the threshold to 1-3X the standard deviation of the full set of tested compounds: 1-3X0.98 = 0.70. Using this threshold, a more manageable 222 compounds were identified as active (dashed line).
(PDF)

S1 Table Canadian Compound Collection (CYCC) Library. (PDF)

S2 Table Pilot screen parameters. (PDF)

S3 Table Top 20 primary bioactives. (PDF)

S4 Table List of compounds selected for experimental pipeline. (PDF)

S5 Table Compound concentration scale (μg/mL) for the MIC analysis. (PDF)

S6 Table Minimal inhibitory concentration (MIC) of the synthetic compounds against *B*. *cenocepacia* K56-2, *P*. *aeruginosa* PA01, *E*. *coli* SY327 and *S*. *aureus* ATCC27700. (PDF)

S7 Table Minimal inhibitory concentration (MIC), Minimal bactericidal concentration (MBC) and Minimal biofilm inhibitory concentration (MBIC) of the top 15 synthetic compounds against ten Bcc panel strains in comparison with *B*. *cenocepacia* K56-2. (XLSX)
