# Supplementary figures and images for: A Pipeline for Screening Small Molecules with Growth Inhibitory Activity against Burkholderia cenocepacia
Source: PLoS One. 2015 Jun 8;10(6):e0128587. doi: 10.1371/journal.pone.0128587 (PMC4460083; doi:10.1371/journal.pone.0128587)

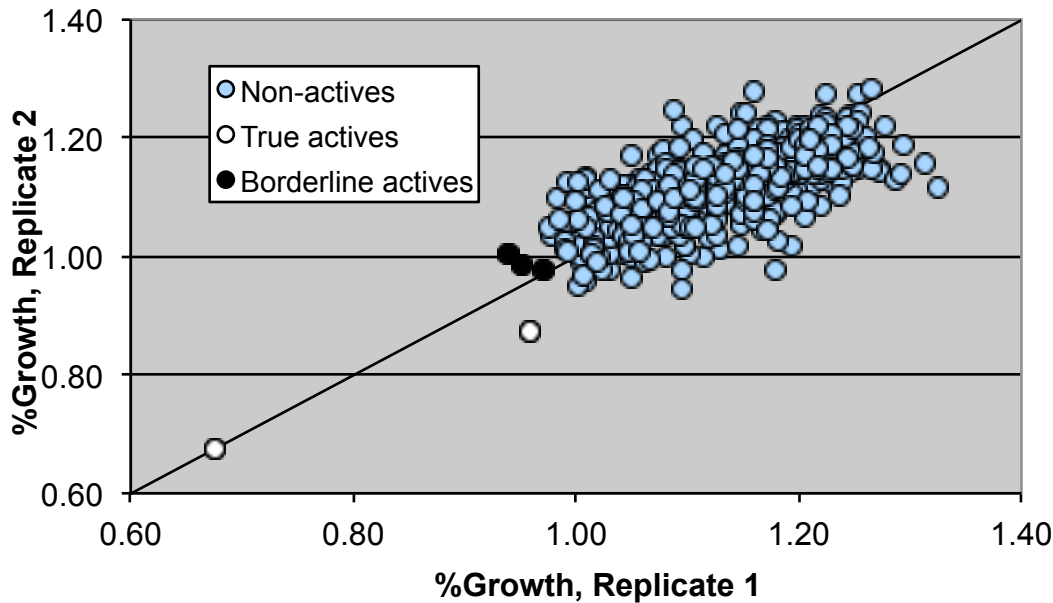

Supplement: S1 Fig — Compounds were tested at 50 μM and 5% DMSO in 96-well format in a final volume of 100 μL. Bacteria was added as a cell suspension obtained from an overnight culture diluted to A 600 of 0.018 in LB. High and low growth controls (8 wells per plate) contained bacteria in LB with 5% DMSO or LB with 5% DMSO, respectively. All assay plates were incubated at 37°C in a humidified incubator. After 5 hr, plates were removed from incubation, sealed with an optically pure seal, and A 600 was measured for 15 s within the absorbance detector (EnVision, Perkin Elmer). In total, 480 compounds were tested in duplicate, with replicates on separate assay plates prepared, and data collected, in tandem. (PDF) [file pone.0128587.s001.pdf]

A

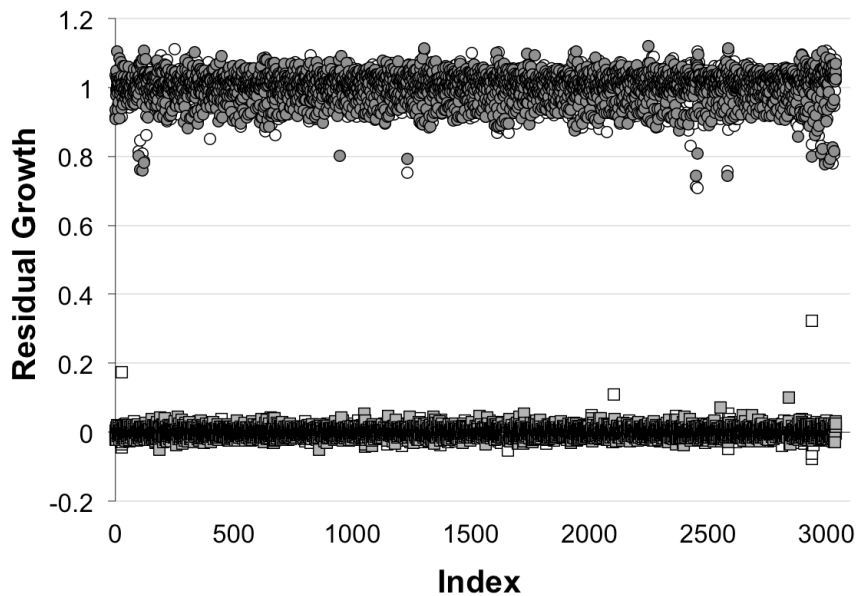

B

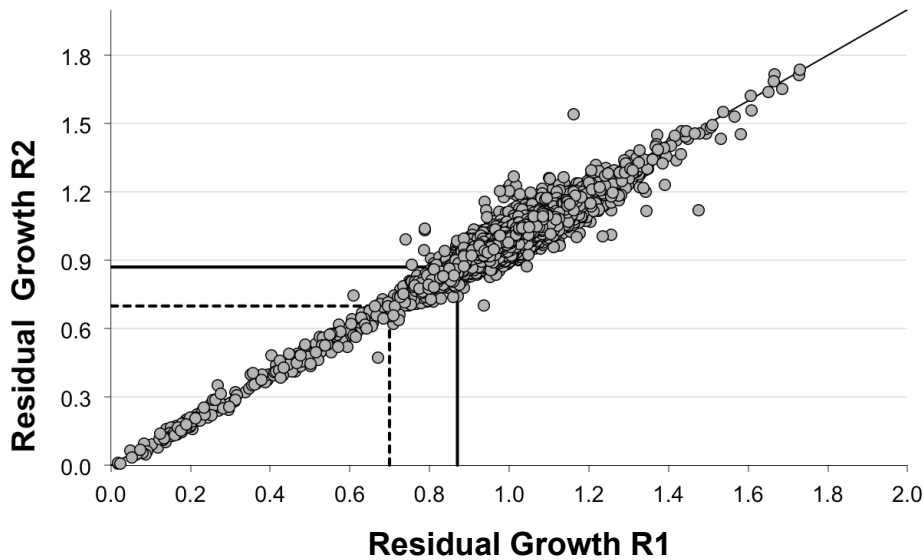

Supplement: S2 Fig — A. B. cenocepacia K56-2 growth in 5% DMSO (high growth controls, circles) were scaled to a residual growth value of 1. Low growth controls (squares) represent media with no bacteria. Controls were run in replicates and replicate 1 (clear symbols) and replicate 2 (Grey symbols) are shown. B. Scatter Plot of the CYCC Library primary screen against B. cenocepacia K56-2 growth Correlation between replicate (R1) and replicate 2 (R2) is shown. Grey circles represent compounds. The statistical cut off rate to identify active compounds was 0.87, as calculated as 1- 3X standard deviation of the high controls, shown as a solid line. This identified 774 actives, a large number of compounds to initially follow up. One method by which to initially focus on the most potent actives is to set the threshold to 1-3X the standard deviation of the full set of tested compounds: 1-3X0.98 = 0.70. Using this threshold, a more manageable 222 compounds were identified as active (dashed line). (PDF) [file pone.0128587.s002.pdf]
