# Supplementary material for: A Pipeline for Screening Small Molecules with Growth Inhibitory Activity against Burkholderia cenocepacia
Source: PLoS One. 2015 Jun 8;10(6):e0128587. doi: 10.1371/journal.pone.0128587 (PMC4460083; doi:10.1371/journal.pone.0128587)
Supplement: S1 Table — (PDF) [file pone.0128587.s003.pdf]

S1 Table. Canadian Compound Collection (CYCC) Library

Subset	Number of compounds	Description
**Custom Library** (Maybridge, Ltd. Cornwall, UK)	16,000	Synthetic small molecules; Average MW = 325 g/mol
**DIVERSet** (ChemBridge Corp., San Diego, CA, USA)	9,989	Synthetic small molecules; Average MW = 325 g/mol
**Prestwick Chemical Library** (Prestwick Chemical, Illkirch, France)	1,120	Off-patent small molecules; >85% FDA approved; Average MW = 325 g/mol
**Natural Products Library** (BIOMOL International, L.P., Plymouth Meeting, PA, USA)	361	Natural products; Average MW = 350 g/mol
**Lopac1280 (International Version)** (Sigma-Aldrich Canada Ltd., Oakville, ON, Canada)	885	Pharmacologically active small molecules; Average MW = 350 g/mol
**Spectrum Collection** (MicroSource Discovery Systems, Inc., Gaylordsville, CT, USA)	1,214	Natural products and bioactives; average MW = 350 g/mol
**Dennis Hall Compounds** Chem. Dept., University of Alberta	690	Synthesized lactams, lactones and piperidines
**Total # compounds**	30,259	
