# Supplementary material for: A Pipeline for Screening Small Molecules with Growth Inhibitory Activity against Burkholderia cenocepacia
Source: PLoS One. 2015 Jun 8;10(6):e0128587. doi: 10.1371/journal.pone.0128587 (PMC4460083; doi:10.1371/journal.pone.0128587)
Supplement: S2 Table — (PDF) [file pone.0128587.s004.pdf]

S2 Table. Pilot screen parameters

	**High Growth Controls**				**Low Growth Controls**			
	Replicate 1		Replicate 2		Replicate 1		Replicate 2	
	A_{600}	% G	A_{600}	% G	A_{600}	% G	A_{600}	% G
**Average**	0.218	1.00	0.221	1.00	0.066	0.00	0.066	0.00
**SD***	0.008	0.05	0.010	0.05	0.002	0.01	0.003	0.01
**Z'***	N/A	0.82	N/A	0.81	N/A	N/A	N/A	N/A
**†Threshold**	N/A	0.86	N/A	0.85	N/A	N/A	N/A	N/A
**Average Threshold**		0.85				N/A		

A_{600}, Absorbance at 600 nm; % G, percent growth
†Threshold = 3 standard deviations of the high growth controls
*See Material and Methods section for calculation methods.
