# Supplementary material for: A Pipeline for Screening Small Molecules with Growth Inhibitory Activity against Burkholderia cenocepacia
Source: PLoS One. 2015 Jun 8;10(6):e0128587. doi: 10.1371/journal.pone.0128587 (PMC4460083; doi:10.1371/journal.pone.0128587)
Supplement: S3 Table — (PDF) [file pone.0128587.s005.pdf]

S3 Table. Top 20 primary bioactives

MAC ID	Average B-Score	Average Residual Growth	Vendor	Name
MAC-0180100	-121.0	0.059	Sigma	Ceftriaxone sodium
MAC-0180630	-109.2	0.230	Sigma	Minocycline hydrochloride
MAC-0181734	-99.1	0.119	MicroSource	Benzalkonium chloride
MAC-0178816	-97.5	0.176	Prestwick	Cefmetazole sodium salt
MAC-0178948	-97.0	0.122	Prestwick	Cefoxitin sodium salt
MAC-0178431	-96.1	0.211	Prestwick	Minocycline hydrochloride
MAC-0178968	-92.0	0.195	Prestwick	Doxycycline hydrochloride
MAC-0179071	-91.2	0.263	Prestwick	Florfenicol
MAC-0178423	-89.0	0.226	Prestwick	Oxytetracycline dihydrate
MAC-0180114	-87.3	0.180	Sigma	Cephapirin sodium
MAC-0183733	-84.2	0.164	D Hall	-
MAC-0178935	-82.6	0.227	Prestwick	Moxalactam disodium salt
MAC-0183726	-82.3	0.167	D Hall	-
MAC-0178351	-80.7	0.078	Prestwick	Metampicillin sodium salt
MAC-0183697	-79.9	0.147	D Hall	-
MAC-0178852	-77.8	0.056	Prestwick	Cefazolin sodium salt
MAC-0036650	-75.9	0.098	Maybridge	-
MAC-0179334	-75.0	0.056	Prestwick	Cefepime hydrochloride
MAC-0178147	-74.3	0.198	Prestwick	Chloramphenicol
MAC-0178337	-73.9	0.183	Prestwick	Norfloxacin
