# Supplementary material for: A Pipeline for Screening Small Molecules with Growth Inhibitory Activity against Burkholderia cenocepacia
Source: PLoS One. 2015 Jun 8;10(6):e0128587. doi: 10.1371/journal.pone.0128587 (PMC4460083; doi:10.1371/journal.pone.0128587)
Supplement: S4 Table — (PDF) [file pone.0128587.s006.pdf]

S4 Table. List of compounds selected for experimental pipeline

MAC ID	PubChem CID	Supplier	Supplier Number
MAC-0000212	2802801	Maybridge	BTB14887
MAC-0002599	5712381	Maybridge	BTB01692
MAC-0004745	2799329	Maybridge	BTB05772
MAC-0004910	2799514	Maybridge	BTB06065
MAC-0012351	2806019	Maybridge	CD11359
MAC-0012675	2806295	Maybridge	DFP00172
MAC-0013209	2806727	Maybridge	DP01299
MAC-0017743	2810852	Maybridge	HTS02734
MAC-0021829	5591254	Maybridge	JFD00020
MAC-0028239	2822094	Maybridge	KM07965
MAC-0029339	2823087	Maybridge	KM09750
MAC-0031247	2826076	Maybridge	PD00301
MAC-0032075	2826653	Maybridge	RDR00047
MAC-0032345	304015	Maybridge	RDR00966
MAC-0033192	2725783	Maybridge	RDR03026
MAC-0036650	5605429	Chembridge	5238276
MAC-0036886	703192	Maybridge	RJC01735
MAC-0040158	2732031	Maybridge	S07624
MAC-0040413	5702402	Maybridge	S10015
MAC-0040599	5702423	Maybridge	S10991

MAC-0040618	5375839	Maybridge	S11082
MAC-0041191	2732974	Maybridge	S14125
MAC-0041192	5702506	Maybridge	S14126
MAC-0041467	275787	Maybridge	S14963
MAC-0044103	2781638	Maybridge	SEW04981
MAC-0044571	5925558	Maybridge	SEW 05753
MAC-0046591	9580786	Maybridge	SPB 00929
MAC-0046850	5804246	Maybridge	SPB01640
MAC-0049900	2746762	Maybridge	SPB07211
MAC-0050194	1380937	Maybridge	SPB07737
MAC-0050919	220986	Maybridge	XBX 00130
MAC-0154923	589423	Chembridge	CD 06845
MAC-0161677	5716375	Chembridge	NRB00390
MAC-0163048	9566292	Maybridge	RF04139
MAC-0164385	697963	Maybridge	S 01396
MAC-0164811	137842	Maybridge	S11897
MAC-0164956	5380390	Maybridge	S14295
MAC-0168816	15400	Maybridge	5133209
MAC-0169562	2832027	Chembridge	5175453
MAC-0169572	2832032	Chembridge	5175529
MAC-0170543	769618	Chembridge	5238271
MAC-0170906	5396175	Chembridge	5258669
MAC-0171133	2838346	Chembridge	5326453

MAC-0171207	819368	Chembridge	5267231
MAC-0172113	2838346	Chembridge	5326453
MAC-0173044	781837	Chembridge	5404961
MAC-0175253	54693803	Chembridge	5626173
MAC-0175391	6743705	Chembridge	5647467
MAC-0177634	6741757	Chembridge	6025732
