# Supplementary material for: A Pipeline for Screening Small Molecules with Growth Inhibitory Activity against Burkholderia cenocepacia
Source: PLoS One. 2015 Jun 8;10(6):e0128587. doi: 10.1371/journal.pone.0128587 (PMC4460083; doi:10.1371/journal.pone.0128587)
Supplement: S5 Table — (PDF) [file pone.0128587.s007.pdf]

Table S5. Compound concentration scale (μg/mL) for the MIC analysis.

Concentration scale		
**0.25-512**[a] (μg/ml)	**0.06-128**[b] (μg/ml)	**0.03-64**[c] (μg/ml)
MAC-0004910	MAC-0000212	MAC-0004745
MAC-0013209	MAC-0028239	MAC-0012351
MAC-0017743	MAC-0029339	MAC-0021829
MAC-0031247	MAC-0044103	MAC-0032075
MAC-0040158	MAC-0046591	MAC-0036650
MAC-0041192	MAC-0046850	MAC-0036886
MAC-0151023	MAC-0161677	MAC-0040413
MAC-0164385	MAC-0164811	MAC-0040599
MAC-0168816	MAC-0169562	MAC-0041191
MAC-0171207	MAC-0169572	MAC-0050194
MAC-0173044	MAC-0172133	MAC-0163048
MAC-0183697	MAC-0175253	MAC-0170543
		MAC-0170906
		MAC-0171133

[a]The standard concentration scale for an MIC as specified by CLSI guidelines in MHB with cation supplementation (CAMHB) is 0.25-512 μg/mL (See material and methods).

[b]The test compounds in this row were soluble in CAMHB when diluted1/40 resulting in an MIC test range of 0.06-128 μg/mL.

[c]The test compounds in this row were soluble in CAMHB when diluted1/80 resulting in an MIC test range of 0.06-128 μg/mL.
