# Supplementary material for: A Pipeline for Screening Small Molecules with Growth Inhibitory Activity against Burkholderia cenocepacia
Source: PLoS One. 2015 Jun 8;10(6):e0128587. doi: 10.1371/journal.pone.0128587 (PMC4460083; doi:10.1371/journal.pone.0128587)
Supplement: S6 Table — (PDF) [file pone.0128587.s008.pdf]

S6 Table.
Minimum inhibitory concentration (MIC) of the synthetic compounds against *B. cenocepacia* K56-2, *P. aeruginosa* PA01, *E. coli* SY327 and *S. aureus* ATCC27700.

		MIC (µg/ml)[a]			
Cnd_ID	Structure	*B. ce* K56-2	*P. ae* PAO1	*E.co* SY327	*S. au* ATCC27700
MAC-0000212	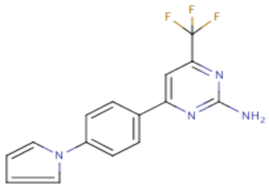	64	>256[*]	>128	>128
MAC-0002599[§]	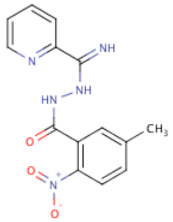	ND	ND	ND	ND
MAC-0004745	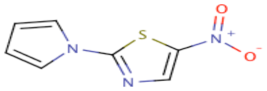	>64	>64[*]	32	>64
MAC-0004910	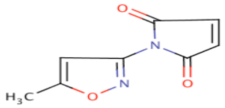	256	256[*]	256	256

MAC-0012351	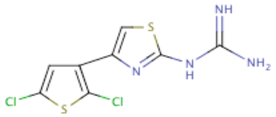	>64	>64*	>64	>64
MAC-0012675[§]	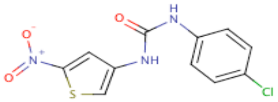	ND	ND	ND	ND
MAC-0013209	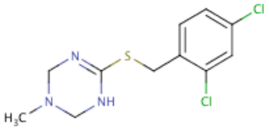	128	256*	256	>512
MAC-0017743	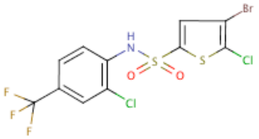	>512	>512*	>512	>512
MAC-0021829	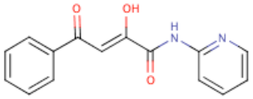	>64	>64*	64	>64

MAC-0028239	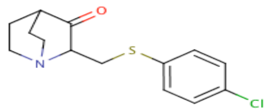	>128	>128*	32	64
MAC-0029339	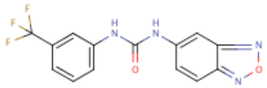	>128	>128*	>128	>128
MAC-0031247	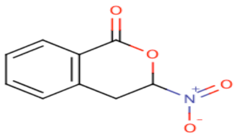	256	512*	256	512
MAC-0032075	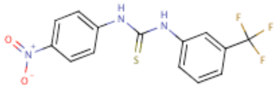	>8	>8*	>64	>64
MAC-0032345§	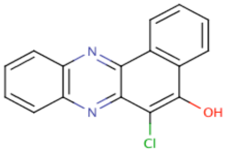	ND	ND	ND	ND
MAC-0033192§	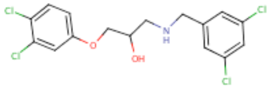	ND	ND	ND	ND

MAC-0036650	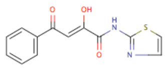	16	>64	16	>64
MAC-0036886	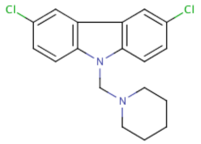	>64	>64*	>64	>64
MAC-0040158	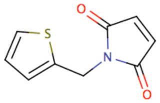	64	256	32	32
MAC-0040413	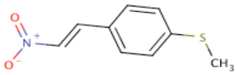	64	>64*	64	>64
MAC-0040599	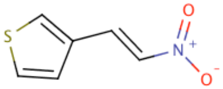	32	64*	64	>64
MAC-0040618§	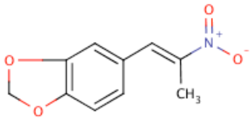	ND	ND	ND	ND

MAC-0041191	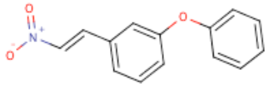	>64	>64*	>64	>64
MAC-0041192	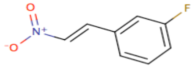	32	64	4	4
MAC-0041467§	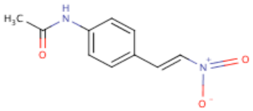	ND	ND	ND	ND
MAC-0044103	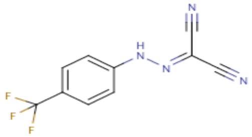	32	>128*	32	128
MAC-0044571§	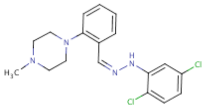	ND	ND	ND	ND

MAC-0046591	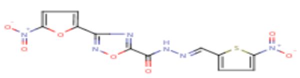	64	>128*	1	8
MAC-0046850	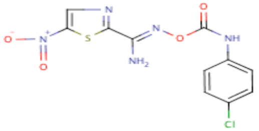	>128	>128*	>128	>128
MAC-0049900§	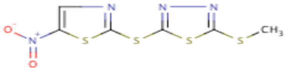	ND	ND	ND	ND
MAC-0050194	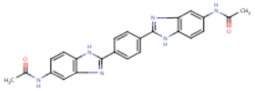	>8	>8*	>64	>64
MAC-0151023	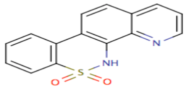	32	512	32	64
MAC-0161677	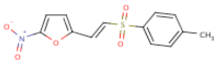	>128	>128*	1	8

MAC-0163048	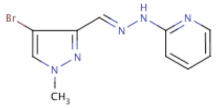	>64	>64*	>64	>64
MAC-0164385	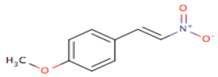	512	>512	>512	512
MAC-0164811	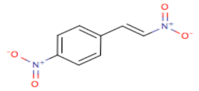	16	>128	8	8
MAC-0164956§	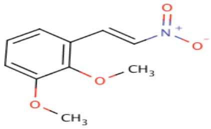	ND	ND	ND	ND
MAC-0168816	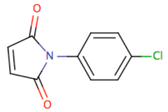	256	>512	256	256
MAC-0169562	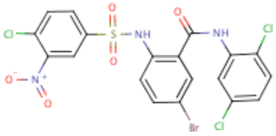	>128	>128*	>128	>128
MAC-0169572	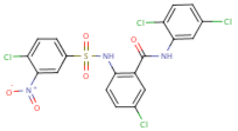	>128	>128*	>128	>128

MAC-0170543	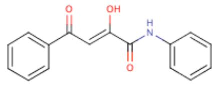	>64	>64*	>64	>64
MAC-0170906	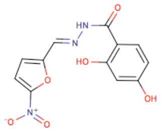	>64	>64	>64	>64
MAC-0171133	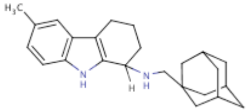	>64	>64*	>64	>64
MAC-0171207	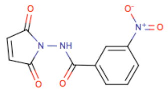	>512	>512	>512	512
MAC-0172113	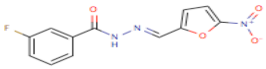	>128	>128	2	16
MAC-0173044	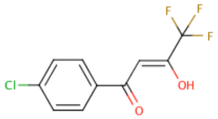	>512	>512*	32	256

MAC-0175253	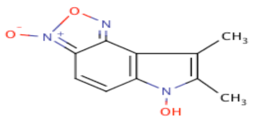	>128	>128[*]	>128	>128
MAC-0175391[§]	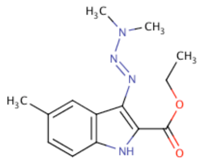	ND	ND	ND	ND
MAC-0177634[§]	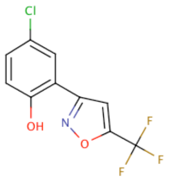	ND	ND	ND	ND
MAC-018697	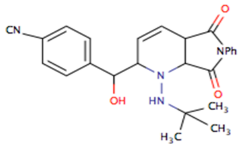	>512	64	64	64

[a]MIC was determined using the standard microtitre broth dilution method as specified by CLSI guidelines in MHB with cation supplementation and a final inoculum of 10^5 CFU/mL. The compound concentration range for the MIC analysis is listed in S5 Table.
[§]These compounds were not soluble at any concentration in CAMHB and were therefore excluded from the MIC analysis.
[*]*P.aeruginosa* strain ATCC27853 was used for the MIC.
